# Reducing model biases is essential to projecting future climate variability

**DOI:** 10.1093/nsr/nwab080

**Published:** 2021-04-30

**Authors:** Toshio Yamagata

Extreme El Niño events not only cause climate disasters leading to enormous socioeconomic losses, but also have devastating impacts on the world's ecosystems [1,2]. A reliable projection of their frequency change in the future warmer climate is therefore very important for our sustainable development as well as disaster prevention [3].

Since the development of extreme El Niño is always accompanied by weakened easterlies and eastward extension of deep convection, a commonly accepted view is that the extreme El Niño, which is defined with convective activities in the Niño3 region, would increase twice in the future warmer climate [4]. Because the sea surface temperature (SST) warming in the eastern tropical Pacific would be faster than its surrounding areas, the climatological zonal and meridional SST gradients, preventing the deep convection from moving eastward in the present-day climate, would be easily reversed by a much smaller-than-today SST anomaly in the eastern tropical Pacific in future.

However, the projected ‘El Niño-like’ SST warming in the tropical Pacific has been often questioned [5] for two main reasons: (i) the winner of the competition between the weakening of the Walker circulation due to the increased atmospheric static stability [6] and the strengthening of easterly winds due to oceanic thermostat mechanism in future [7,8] is still unknown [5,9]; and (ii) the remarkably distinct tropical Pacific SST trend between model simulations and observations over the past [10–13] highlights the existence of systematic model biases in CMIP5. Accordingly, a key question is raised: Would the tropical eastern Pacific warm faster than its surrounding areas?

In this issue, Tang *et al.* [14] make it clear for the first time that models’ common biases may have great impacts on the projection of future tropical Pacific SST change. By identifying 13 common biases in simulating the past climate, they suggest the SST warming in the tropical eastern Pacific may be largely overprojected. Surprisingly, the SST change after correcting the impacts of the models’ common biases shows that the strongest warming occurs in the tropical western Pacific rather than in the east, accompanied by stronger easterlies and suppressed convection in the eastern Pacific. This shows a stark contrast to the original CMIP5 projection.

**Figure 1.**
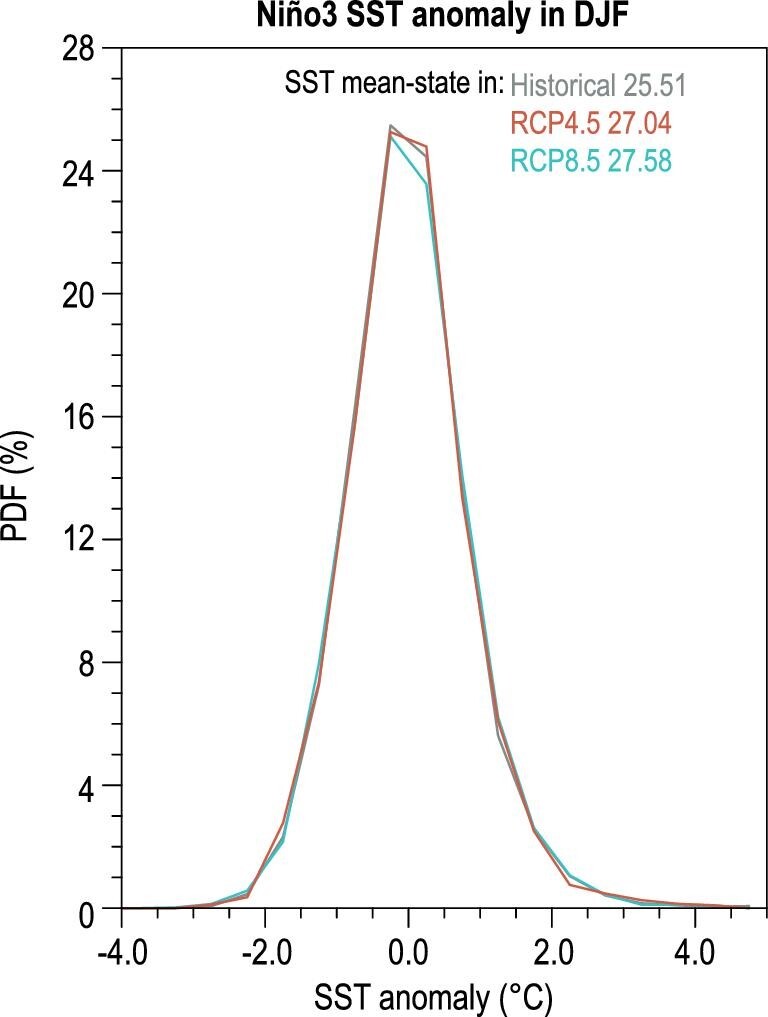
The probability density distribution of Niño3 SST anomaly in boreal winter. The gray, red and blue curves denote the results based on the historical simulations, RCP4.5 scenario and RCP8.5 scenario, respectively. The multimodel ensemble means of Niño3 SST climatology in the three experiments are also displayed. Results are produced based on the 28 CMIP5 models analyzed in Ref. [14]. PDF: probability density distribution. DJF: December, January and February. SST: sea surface temperature.

Interestingly, Tang *et al.* [14] suggest the originally projected two-fold increase of extreme El Niño is mostly determined by the mean-state change, while the anomaly itself would not change much. This view is supported well by the almost identical probability density distribution of Niño3 SST anomaly (Fig. 1) Therefore, the result indicates that the originally projected two-fold change in the extreme El Niño frequency defined by total values may be largely due to the models’ common biases in the projection of mean-state changes. Even though the complex interactions between mean state and El Niño/Southern Oscillation may influence such a statistical correction, the results of Tang *et al.* [14] assert that a reliable projection of the future extreme El Niño frequency change requires the correct mean-state projection. Indeed, Zhao and Fedorov [15] have recently suggested that the ENSO would be suppressed due to the enhanced east-minus-west SST gradient, associated with the strengthened easterlies.

In summary, the findings of Tang *et al.* [14] shed a new light on the importance of correcting systematic biases before getting reliable projection on future climate variability. More efforts are certainly necessary to reduce climate models’ common biases in coming years.

***Conflict of interest statement*.** None declared.
